# Sleep deprivation impairs neurovascular coupling and cerebral vasomotor reactivity

**DOI:** 10.1038/s41598-025-94212-w

**Published:** 2025-03-19

**Authors:** Krisztina Szonja Rab-Bábel, Dóra Sulina, Cecilia Daniel, Tibor Rab, Norbert Kozák, László Oláh

**Affiliations:** Department of Neurology, Faculty of Medicine, University of Debrecen, Móricz Zsigmond Str. 22., 4032 Debrecen, Hungary

**Keywords:** Sleep deprivation, Cerebral blood flow, Transcranial doppler, Vasoreactivity, Neurovascular coupling, Neuroscience, Neuro-vascular interactions

## Abstract

Sleep deficiency increases the risk of cerebrovascular diseases. However, the effects of sleep deprivation (SD) on cerebral blood flow have been poorly studied. We examined the effect of 24-h of SD on the resting posterior cerebral artery (PCA) and middle cerebral artery (MCA) flow velocities (FV), the visually evoked FV response in the PCA (neurovascular coupling), and the hypercapnia-induced FV response in the MCA (cerebral vasoreactivity). Visual evoked potential (VEP) and transcranial Doppler examinations were performed in 25 healthy adults before and after 24-h of SD. Cerebral vasoreactivity was measured by breath-holding test in left and right MCA. The visually evoked FV response was evaluated in left and right PCA. There was a tendency for increased resting mean FV in PCA (*p* = 0.08) and MCA (*p* = 0.07) after SD. Both the visually evoked FV response in the PCA and the hypercapnia-induced FV increase in the MCA were significantly lower after than before SD, however, no change in VEP amplitudes was found. Our study suggested that the impaired functional stimulation-evoked FV response after SD was not due to a reduced neuronal activation, but probably to a decreased vasodilatory response. Negative effects of SD on cerebral hemodynamics were also demonstrated by reduced cerebral vasoreactivity.

## Introduction

Due to changes in social and living conditions more and more people sleep less than required, and chronic insomnia affects approximately 10% of the population^1^. According to the consensus recommendation of the American Academy of Sleep Medicine (AASM) and Sleep Research Society (SRS) „an adult should sleep 7 h or more per night on a regular basis to promote optimal health”^2^. The age-adjusted prevalence of US adults who reported short sleep duration was 34.8% in 2020^3^.

There is a strong evidence of association between sleep quality and quantity and health. One-night sleep deprivation (SD, i.e. total lack of sleep for one night) has detrimental effects on the cognitive functions, including visual perception, attention, working memory, and executive control tasks^4–6^. Sleep deficiency, however, may affect not only the mental but also the physical health. Sleep restriction was shown to impair glucose homeostasis and insulin sensitivity, and enhance the release of norepinephrine^7,8^ and secretion of inflammatory cytokines^7^. These proinflammatory, hormonal and metabolic changes, together with the altered arterial baroreflex and endothelial dysfunction, which were also reported in patients with sleep deficiency, lead to an increased risk of obesity, hypertension, coronary artery disease and cerebrovascular diseases^7^. Previous animal studies have suggested that SD also influences the cerebral blood flow regulation through vessel expansion and decreased vascular compliance^9,10^.

As brain has no glucose and energy reserves, a continuous supply of blood and oxygen is essential for its function. While autoregulation assures a relatively constant cerebral blood flow in a wide range of blood pressure, the local cerebral blood flow is finely tuned to dynamic changes of neuronal activity. This phenomenon is called neurovascular coupling or functional hyperaemia that serves for matching the additional demand of nutrients and removal of heat and waste poducts in an activated brain tissue by increased regional cerebral blood flow^11^. Several methods are available to investigate the neurovascular coupling, including functional magnetic resonance imaging, functional near-infrared spectroscopy, and functional transcranial Doppler (TCD). All these functional examinations are based on the same phenomenon: neuronal activation is coupled to an increase of regional cerebral blood flow that can be measured with use of different techniques^12–14^.

Although the effect of SD on cerebral hemodynamics is poorly investigated, some animal and human studies showed that extended brain activity through SD caused vasodilation of cerebral resistance vessels^10^ and increase in cerebral blood flow^15^. Moreover, decreased vascular compliance indicated by blunted neuronal activation-induced vascular response after SD was also found in animal experiments^9,10^ and in one human study^16^. Animal studies suggested that vasodilation during SD may reach its capacity, limiting blood delivery, and leading to metabolic deficits with the potential for neuronal damage^9,10^. However, the above studies investigating the effect of SD on cerebral hemodynamics left some questions unanswered. The animal studies could detect decreased auditory evoked flow response after SD only during the recovery sleep, but not during awake state. Furthermore, the only human study that reported blunted task-induced flow response after 24-h of SD^16^ could detect reduced neurovascular coupling related hemodynamic changes when measured by fNIRS in the prefrontal cortex, but no significant decrease of task-induced flow response could be found by functional TCD and dynamic retinal vessel analysis. In addition to the inconsistent results, the question remained unanswered whether the impaired task-induced hemodynamic response was a consequence of the dysfunction of supplying blood vessels, neuronal activation, or neurovascular coupling itself. Since SD impairs cognitive functions, it is important to know whether lack of sleep has a direct effect on neuronal activity, or it exerts its detrimental effect indirectly by affecting cerebral circulation. In order to answer this question we separately assessed the stimulus-induced neuronal activity and the neuronal activity-evoked flow response before and after 24-h acute total SD. Moreover, the hypercapnia-indued flow velocity (FV) response was also examined as a different pathway to test the vasodilatory properties of cerebral vessels independent of neuronal activation.

In the present study, TCD was used to investigate the cerebral hemodynamics. TCD has a poor spatial resolution, but its temporal resolution is excellent and it provides a powerful tool for non-invasive and repeated assessment of cerebral hemodynamics. By measuring FV changes, TCD can assess cerebral vascular responses to various vasodilatory stimuli, including neuronal activation and hypercapnia. Changes in FV in response to neuronal activation are referred to as neurovascular coupling, and those due to changes in partial pressure of carbon dioxide or to administration of acetazolamide are referred to as cerebral vasoreactivity. It should be noted that these CBF regulatory mechanisms are regulated at the level of the cerebral resistance vessels (arterioles and other microvessels) with no, or with minimal changes of the diameter of the insonated large cerebral arteries^17–19^. Although the regulation of the hypercapnia- and neuronal activation-induced flow response is at least partially different^20,21^, both stimuli result in vasodilation of the resistance vessels leading to a decrease in vascular resistance and an increase in flow and FV in the large cerebral arteries. Supposing that the diameter of the insonated large cerebral artery does not change in response to stimulus that affects cerebral microcirculation^17–19^, changes of cerebral blood FV in the intracranial arteries are proportional to the changes of regional cerebral blood flow, within one individual^22^. Vascular resitance in the supplying area of the insonated vessel can also be evaluated by a TCD parameter called pulsatility index (PI).

Pattern-reversal checkerboard stimulation was applied to examine the neuronal activation-evoked hemodynamic response in the PCA, and the same visual stimulation technique was used to assess the neuronal activation through visul evoked potential (VEP) amplitudes before and after SD. VEP amplitude was used to measure the degree of neuronal activation, while the PCA flow velocity changes reflected the hemodynamic response induced by neuronal activation. Using visual stimulation paradigm and monitoring visually evoked FV parameters in the PCA have several advantages compared to other stimulation methods leading to MCA FV changes. First, visual stimulation causes a robust increase in PCA FV (approximately 15–30% depending on the complexity of the visual stimulus), which is much higher than the FV increase in the appropriate MCA during finger movement (≈10%) or speech (≈5%)^23^. Second, in the PCA territory, the visual function determines the flow response; however, in the MCA territory, many functions (movement, sensory stimulus, thinking, hearing, and speaking) may influence it. Third, the effect of visual stimulus on neuronal activity can easily be assessed by VEP recordings, while the neuronal activation during other stimuli leading to an increase in MCA FV cannot be easily evaluated^24^.

In the present study, we examined the effect of 24-h acute total SD on the resting cerebral blood FV in the right and left middle and posterior cerebral arteries, the latency and amplitude of the visually evoked potential over the right and left visual cortex, and the visual stimulation-evoked posterior cerebral artery (PCA) FV response in healthy subjects. In addition to examining the neurovascular coupling, hypercapnia-induced FV changes were also measured using the breath-holding test in the right and left middle cerebral arteries to assess the effect of total SD on vascular response independent of neuronal activation. We sought to answer, whether 24-h of total SD impairs the neuronal activation-induced flow response, and if so, whether it can be explained by decreased neuronal activity. By separately assessing the hypercapnia-induced vascular response, we tested the effects of SD on cerebral vasomotor reactivity, another CBF regulatory mechnism that also requires vasodilation, but independent of neuronal activation. To the best of our knowledge, this is the first human study to examine the effect of 24-h total SD on the hypercapnia-induced cerebral vasomotor reactivity and to combine the assessment of the neuronal activation-induced flow response with the evaluation of the neuronal activity before and after SD.

## Materials and methods

### Study participants

Twenty-five young, healthy adults (11 males and 14 females), between 21 and 38 years of age (mean age 28 ± 8 years) were included in the study. The study was approved by the Ethics Committee of the Hungarian Medical Research Council (Registration number: 26425–8/2018/EÜIG) and was conducted according to the guidelines of the Declaration of Helsinki. All the participants gave informed consent to participate in the research.

Subjects with cerebrovascular risk factors such as smoking, arterial hypertension, diabetes mellitus, extreme obesity (body mass index higher than 35 kg/m^2^), alcohol dependency, history of migraine, coronary, or peripheral artery diseases, and infection within 1 month of the examination date were excluded. The study protocol included a complete neurological and ophthalmological examination, carotid and vertebral artery duplex ultrasound, and routine clinical laboratory tests (serum ions, creatinine, fasting glucose, hepatic enzymes, creatinine kinase, serum lipids, and inflammatory markers). Blood was drawn after an overnight fast between 7 and 9 a.m., one day before the first examination (day 0). The menstrual cycle phase of female participants was not measured in our self-control study, as any potential sexual hormonal fluctuations between the pre- and post-SD assessments were considered negligible due to the short 24-h interval between the control (pre-SD) and test (post-SD) measurements.

### Experimental design: study protocol

All participants were asked to keep a sleep diary for at least 5 days prior to the day of sleep deprivation (SD). None of the participants reported sleep disturbances. Examinations before and after SD were performed on 2 consecutive days: the control examinations were performed in the morning of the 1st day after a night of sleep (day 1), while test examinations were performed in the morning of the 2nd day after a 24-h SD (day 2). Participants were asked to refrain from alcohol and caffeine consumption for at least 12 h before the examinations. Both the control and test examinations were performed in the morning on fasting state. Blood pressure and pulse rate were measured noninvasively at the beginning and at the end of the experiments on both days.

The examinations on day 1 (control examinations) and day 2 (test examinations) were performed in the same order. First, the visual stimulation-evoked flow velocity (FV) response was measured by transcranial Doppler (TCD) in left and right PCA. Afterwards, cerebral vasoreactivity was investigated by analysing the effect of 30 s breath-holding on the mean FV increase (BHI) in left and right MCA. Finally, visual evoked potentials were recorded (Neuron-Spectrum-4/EPM, Neurosoft, Ivanovo, Russia).

### Functional TCD study

Two 2 MHz probes were fixed by an individually fitted headband over the temporal cranial window. For evaluation of neurovascular coupling, the P2 segment of the PCA was insonated on both sides at a depth of 58–60 mm. Peak-systolic (PSV) and time averaged mean (TAMV) blood flow velocities and pulsatility indices were recorded with a Multidop T Doppler device (DWL, Singen, Germany). For measurement of vasoreactivity, the M1 segment of the MCA was insonated bilaterally at a depth of 50 mm. The procedure for finding and identifying the vessels followed the description of Fujioka and Donville^25^ for the transtemporal approach. Considering that a vasodilator stimulus has a greater effect on mean FV values, but peak-systolic FV is less influenced by Doppler artefacts^26^, we recorded and analysed both velocity indices.

**Fig. 1:**
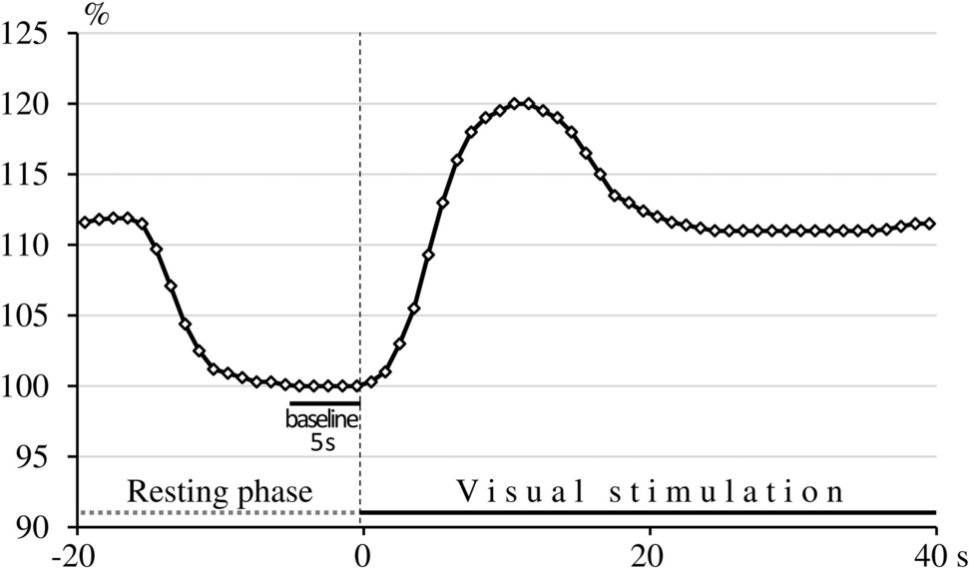
Schematic figure of the visually evoked flow response in the posterior cerebral artery. With a short time delay at the beginning of the visual stimulation (0 s), relative cerebral blood flow velocity increases rapidly, overshoots, and then stabilizes at a constant but higher level than the baseline.

Neurovascular coupling was evaluated using a visual stimulation paradigm. As a stimulation paradigme used a high-contrast, black-and-white checkerboard pattern-reversal stimulation, with a 32 × 32 checkerboard pattern and 2 Hz reversal frequency, generated on a computer screen. Visually evoked FV response during visual stimulation was measured in left and right PCA. The stimulation protocol consisted of 10 cycles, with a resting phase of 20 s and a stimulation phase of 40 s for each cycle. In the resting phases, the subjects closed their eyes; in the stimulation phases, they were instructed to focus their gaze on a red dot at the centre of the checkerboard. Within one person, FV data and pulsatility indices of 10 cycles were averaged. Beat-to-beat intervals of cerebral blood FV data and pulsatility indices were interpolated linearly with a “virtual” time resolution of 10 ms for averaging procedures. To ensure independence from the insonation angle and to allow comparisons between volunteers, absolute data were transformed into relative changes in cerebral blood FV and pulsatility indices in relation to baseline. Baseline was calculated from the blood FV and pulsatility indices averaged for a time span of 5 s at the end of the resting phase, before the beginning of the stimulation phase. With a short time delay at the beginning of the visual stimulation, cerebral blood FV increased rapidly, overshot, and then stabilized at a constant but higher level than the baseline (Fig. 1). To analyse the maximum increase in relative FV changes, the highest of the relative values obtained during the stimulation phase was taken from each subject. Pulsatility indices changed in the opposite direction. To determine the maximum decrease in pulsatility indices, the lowest of the relative values during the stimulation phase was used. Relative flow velocities and pulsatility indices were expressed as a percentage of baseline.

### Breath-holding index

Cerebral vasoreactivity was investigated by analysing the effect of breath-holding on the FV increase in left and right MCA (breath-holding test)^27,28^. At the end of a deep inspiration, subjects were asked to hold their breath for a period of 30 s. Participants were taught not to perform Valsalva manouver during breath-holding. Systolic and mean blood flow velocities in the MCAs were recorded before (the baseline) and during the breath-holding period. The breath-holding index (BHI) was calculated by dividing the percent increase in mean FV by the duration of the breath-holding period (30 s)^29^. The BHI on the two sides was averaged, and the averaged data were used for analysis.

### Visual-evoked potential

For visual evoked potential recordings from the scalp, standard 10-mm silver/silver chloride cup electroencephalography electrodes were used, fixed by contact paste. Three active electrodes were placed according to the recommendations of the International Society for Clinical Electrophysiology of Vision (ISCEV): the Oz electrode was placed 10% of the inion-nasion distance above the inion, and the O1 and O2 electrodes were placed 10% of the head circumference laterally to the left and right, respectively. The Fz reference electrode was placed 20% of the inion-nasion distance anterior to the vertex. The ground was placed at the vertex (Cz). The filter setting was 1–100 Hz. The analysis time was 600 ms, and the sensitivity was 10 mV per division. VEPs at three montages (Oz–Fz, O1–Fz and O2–Fz) were recorded. Two hundred responses evoked by checkerboard pattern reversal stimulation were averaged (Neuron-Spectrum-4/EPM, Neurosoft, Ivanovo, Russia), and latencies and amplitudes of the P100 waves were calculated.

The TCD and VEP examinations were performed in a quiet room at about 22°C while the subjects were sitting comfortably. TCD examinations were always performed by the same examiners.

### Statistical analysis

Data were expressed as means ± standard deviation. The absolute and relative FV data, absolute and relative pulsatility indices, cerebral vasoreactivity values, and VEP latencies and amplitudes, measured on the right and left sides were averaged, and the averaged data were used for analysis.

The Shapiro–Wilk test was used to evaluate whether the continuous variables were normally distributed. Blood pressure values, heart rates, VEP P100 amplitudes and latencies, absolute baseline flow velocities and pulsatility indices, absolute maximum FV values and pulsatility indices, maximum increase in relative flow velocities and maximum decrease in pulsatility indices before and after SD were compared by paired *t*-test. A simple regression analysis was performed to test the relationship between neuronal activation-induced FV changes (maximum relative PSV and maximum relative TAMV) and BHI.

Repeated measures analysis of variance (ANOVA) was applied to compare relative changes in cerebral blood flow velocities and pulsatility indices in the stimulation phases before and after SD. The results of the repeated measures analysis of variance were shown by the group main effect and the group with time-of-measurement interaction. The group main effect showed whether there was a significant difference in flow velocities averaged over the 40-s active period during visual stimulation before and after SD. The group with time-of-measurement interaction indicated whether the pattern of FV or pulsatility index changes over time was different before and after SD. A non-significant interaction indicated that the FV or pulsatility index time courses before and after SD were parallel.

A difference of *p* ≤ 0.05 was considered statistically significant.

## Results

No clinically significant differences were found in the laboratory data in any study subject. The systolic blood pressure (116 ± 6 Hgmm vs 115 ± 7 Hgmm, *p* = 0.69), diastolic blood pressure (75 ± 5 Hgmm vs 74 ± 5 Hgmm, *p* = 0.68), and pulse rate (71 ± 7 min^−1^ vs 70 ± 5 min^−1^, *p* = 0.72) recorded at the beginning of the measurements before and after sleep deprivation (SD) were comparable. In three volunteers only unilateral recording of PCA flow parameters could be performed due to technical reasons. In these volunteers the unilateral PCA flow data were used for the statistical analysis. In one volunteer none of the TCD probes could be fixed to insonate the PCA flow, therefore the visually evoked flow parameters were based on data of 24 subjects. Demographics, sleep-related data, and laboratory values are shown in Table 1.

**Table 1:** Demographic, sleep and laboratory data.

Parameters	Values (mean ± standard deviation)
**I. Demographic data**	
Height (cm)	170.6 ± 7.7
Body weight (kg)	68.8 ± 13.4
BMI (kg/m^2^)	23.6 ± 4.4
Age (years)	28 ± 8
Sex	Males 44%, Females 56%
**II. Sleep data**	
Average hours of sleep/night	7.4 ± 0.5
Average numbers of awakenings/last 7 nights	1.8 ± 0.9
**III. Laboratory parameters**	
Sodium (mmol/L)	139.1 ± 1.9
Potassium (mmol/L)	4.2 ± 0.3
Chloride (mmol/L)	104 ± 2.4
Glucose (mmol/L)	5.3 ± 0.9
Blood urea nitrogen (mmol/L)	4.5 ± 1.0
Creatinine (μmol/L)	68.3 ± 16.4
GFR (mL/min/1.73m^2^)	88.8 ± 3.5
AST (U/L)	21.6 ± 11.3
ALT (U/L)	22.5 ± 11.1
GGT (U/L)	33.8 ± 29.6
Creatinine Kinase (U/L)	121.9 ± 82.1
LDH (U/L)	199 ± 34.7
CRP (mg/L)	4.1 ± 6.9
Triglyceride (mmol/L)	1.7 ± 0.9
Total Cholesterol (mmol/L)	5.1 ± 1.1
WBC (10^9^/L)	7.3 ± 1.7
RBC (10^12^/L)	4.8 ± 0.5
Hemoglobin (g/L)	145.5 ± 12.2
Platelets (10^9^/L)	222.3 ± 64.7

BMI: body mass index; GFR: glomerular filtration rate; AST: aspartate transaminase; ALT: alanine transaminase; GGT: gamma-glutamyl transferase; LDH: lactate dehydrogenase; CRP: C-reactive protein; WBC: white blood cell; RBC: red blood cell. Data are mean ± standard deviation.

### Effect of sleep deprivation on cerebral blood flow velocity: baseline values and visually evoked flow parameters

At first, baseline absolute peak systolic flow velocities recorded in the PCA, and time-averaged mean flow velocities, and pulsatility indices (PI) measured in the PCA and MCA before and after SD were compared (Table 2.). Blood FV parameters recorded for a time span of 5 s at the end of the resting phase in the PCA were considered baseline (Fig. 1). No statistically significant differences were found in the baseline TAMV and PSV or PI measured in the PCA before and after SD, however, a tendency towards higher flow velocities (*p* = 0.16 for PSV; *p* = 0.08 for TAMV) and lower PI (*p* = 0.06) after SD compared to the control period could be detected (Table 2). Similar to PCA, there was also a trend in the MCA towards higher resting TAMV (*p* = 0.07) and lower PI (*p* = 0.07) after SD (Table 2).

**Table 2:** Absolute flow velocity parameters in the resting phase (baseline) and during visual stimulation (maximum absolute flow velocity and minimum pulsatility index values), and maximum changes of absolute flow velocity and pulsatility index values during visual stimulation compared to baseline, before and after sleep deprivation (SD).

Parameters	Before SD (control period)	After SD (test period)	p value
**Baseline absolute values at the resting phase**			
Baseline MCA TAMV (cm/s)	55.34 ± 9.70	58.31 ± 11.29	0.07
Baseline MCA PI	0.78 ± 0.14	0.74 ± 0.09	0.07
Baseline PCA PSV (cm/s)	47.82 ± 8.88	51.40 ± 7.81	0.16
Baseline PCA TAMV (cm/s)	31.12 ± 6.11	34.16 ± 6.01	0.08
Baseline PCA PI	0.84 ± 0.12	0.79 ± 0.14	0.06
**Maximum absolute flow velocity or minimum pulsatility index values during visual stimulation**			
Maximum PCA PSV (cm/s)	55.78 ± 9.91	59.09 ± 8.53	0.19
Maximum PCA TAMV (cm/s)	38.03 ± 7.41	40.83 ± 6.65	0.12
Minimum PCA PI	0.71 ± 0.09	0.69 ± 0.10	0.42
**Maximum changes of absolute flow velocities and pulsatility index during visual stimulation**			
Maximum—baseline PCA PSV (cm/s)	7.96 ± 2.05	7.69 ± 2.30	0.66
Maximum—baseline PCA TAMV (cm/s)	6.91 ± 1.88	6.68 ± 2.33	0.73
Minimum—baseline PCA PI	-0.13 ± 0.04	-0.10 ± 0.05	< 0.01

MCA: middle cerebral artery; PCA: posterior cerebral artery; PI: pulsatility index; PSV: peak-systolic flow velocity; SD: sleep deprivation; TAMV: time-averaged mean flow velocity. Data before and after SD were compared with paired t-test. Data are mean ± standard deviation.

Comparing the maximum absolute PSV, TAMV and minimum absolute PI values during the visual stimulation before and after SD, paired-t test did not reveal significant difference (Table 2). The absolute FV changes during the visual stimulation before and after SD were not significant either. However, the decrease in absolute PI during the visual stimulation was significantly smaller after SD than before SD (p < 0.01; Table 2).

**Fig. 2:**
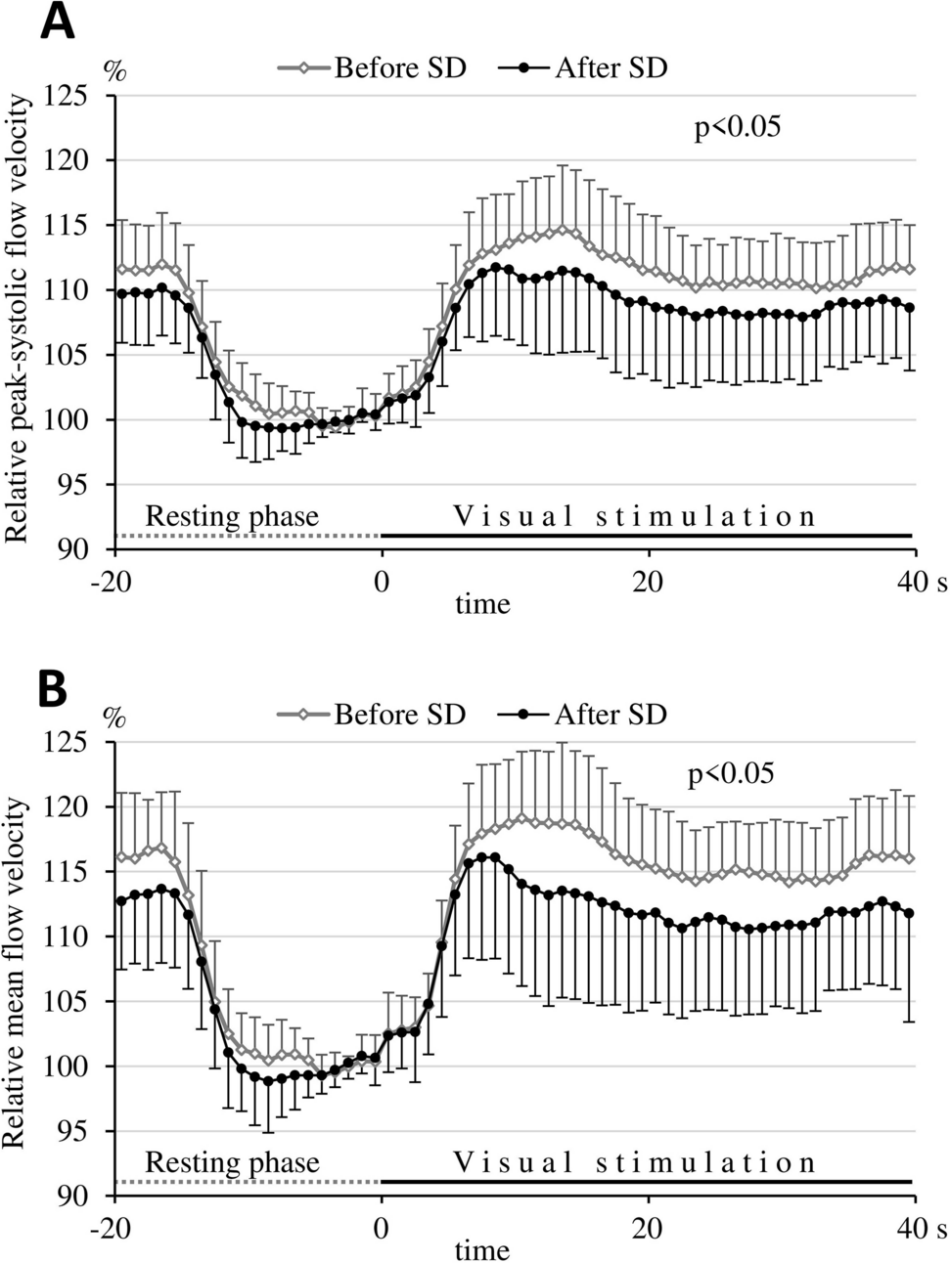
Relative peak systolic (**A**) and mean flow velocity (**B**) time courses before and after sleep deprivation. In order to avoid overlapping of the SD bars and to improve the perspicuity of the figure, standard deviation bars are upward on the curves “before SD”, while they are downward on the curves “after SD”. Note that the increase in both the relative peak systolic and mean flow velocities during the visual stimulation was larger before sleep deprivation than after sleep deprivation. Repeated measures analysis of variance was applied to compare relative changes in cerebral blood flow velocities in the stimulation phases before and after sleep deprivation; group main effect: *p* < 0.05.

Relative FV values measured during the visual stimulation before and after SD were calculated in relation to the proper baseline values. Testing for the effect of neuronal activation on relative changes in FV and pulsatility index showed an increase in flow velocities and a decrease in PI during visual stimulation compared to baseline, either before or after SD (Fig. 2).

Analyzing the visually evoked relative FV time courses between the control (before SD) and test (after SD) periods, repeated measures ANOVA showed a significant group main effect (p < 0.05 for both the PSV and TAMV values), indicating that the relative flow velocities during visual stimulation were smaller after SD than in the control period, i.e., before SD (Fig. 2A, B). Smaller relative flow velocities during SD mean that the flow velocities evoked by visual stimulation increased less after 24 h of SD compared to the control period. The group with time of measurement interaction was also significant in the mean FV values (*p* < 0.01), which means that the pattern of FV changes was different before and after SD.

**Fig. 3:**
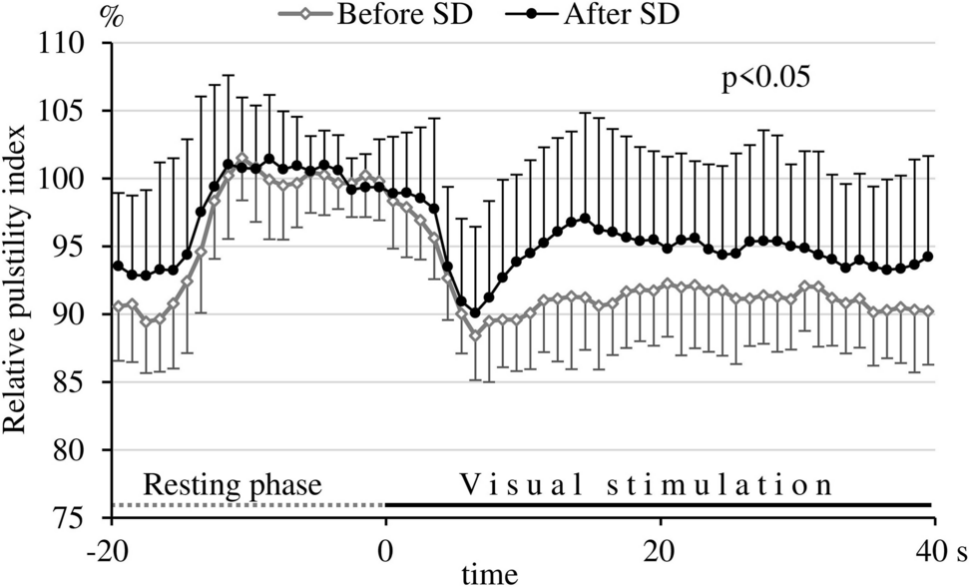
Relative pulsatility index time courses before and after sleep deprivation. In order to avoid overlapping of the SD bars and to improve the perspicuity of the figure, standard deviation bars are upward on the curves “after SD”, while they are downward on the curves “before SD”. Note that the decrease in pulsatility indices during the visual stimulation was larger before sleep deprivation than after sleep deprivation. A smaller decrease in the pulsatility index induced by visual stimulation after SD indicates a smaller decrease in vascular resistance (i.e., less pronounced vasodilation) in the territory of the posterior cerebral artery. Repeated measures analysis of variance was applied to compare relative changes in pulsatility indices in the stimulation phases before and after sleep deprivation; group main effect: p < 0.05.

Repeted measures ANOVA revealed a significant group main effect (p < 0.05) and a significant group with time of measurement interaction (p < 0.01) also in the visually evoked relative pulsatility index time courses (Fig. 3) between the control (before SD) and test (after SD) periods. Higher relative pulsatility indices during visual stimulation indicate that the decrease in pulsatility indices induced by visual stimulation was significantly smaller after SD than before SD.

Analysis of maximum changes in the relative flow velocities and relative PI showed that the maximum decrease of PI (*p* = 0.04) and maximum increase of PSV (*p* = 0.01) during visual stimulation were significantly smaller after SD than before SD, but no significant difference (*p* = 0.19) was found in the maximum increase of TAMV between the test and control periods (Table 3).

**Table 3:** Maximum relative flow velocities and minimum relative pulsatility indices measured in the posterior cerebral artery during visual stimulation, before and after sleep deprivation (SD).

Parameters	Before SD (control period)	After SD (test period)	*p*
Maximum relative PSV (%)	117.94 ± 3.53	115.52 ± 4.78	0.01
Maximum relative TAMV (%)	122.51 ± 4.46	120.83 ± 7.39	0.19
Maximum relative PI (%)	83.80 ± 2.59	86.24 ± 5.61	0.04

PSV: peak systolic flow velocity; TAMV: time-averaged mean flow velocity; PI: pulsatility index. SD: sleep deprivation. Data before and after SD were compared with paired t-test. Data are mean ± standard deviation.

### Effect of sleep deprivation on neuronal activity: VEP parameters

All participating subjects’ visual acuity was 1.0 on both sides. After SD, the latency of the VEP P100 wave increased (before SD 108.93 ± 3.90 ms vs. after SD 111.98 ± 4.46 ms, *p* < 0.01), whereas the amplitude of the VEP P100 wave did not change (before SD 9.80 ± 6.21 μV vs. after SD 9.04 ± 4.86 μV, *p* = 0.26) compared to the control pre-SD period.

### Effect of sleep deprivation on cerebral vasoreactivity: breath-holding index parameters

The increment of blood FV in the MCA caused by 30 s of breath-holding was lower after than before SD (before SD 1.63 ± 0.47%/s vs. after SD 1.42 ± 0.43%/s, *p* = 0.023). Simple regression analysis did not show significant relationship between BHI measured in the MCA and visual stimulation-induced maximum relative TAMV changes (r = 0.009, *p* = 0.96 before SD; r = 0.1, *p* = 0.64 after SD) or maximum relative PSV changes (r = 0.158, *p* = 0.46 before SD; r = 0.153, *p* = 0.47 after SD) in the PCA.

## Discussion

In the present study, we examined the effect of sleep deprivation (SD) on the resting FV (flow velocity) and PI (pulsatility index) in the PCA (posterior cerebral artery) and MCA (middle cerebral artery), the occipital cortex activation caused by visual stimulation, the visual stimulation-evoked flow response in the PCA, and the hypercapnia-induced cerebral vasomotor reactivity in the MCA. After 24 h of sleep deprivation, we found a tendency towards higher resting FV and smaller PI in both the MCA and PCA suggesting that SD caused cerebral vasodilation and decrease in the basal cerebrovascular tone. We also showed that despite no change in VEP amplitude, the visual stimulation-evoked FV and PI changes in the PCA were smaller after 24-h of SD than in the control period, before SD. Moreover, not only the neuronal activation-induced hemodynamic response in the PCA, but also the hypercapnia-induced vasomotor reactivity in the MCA was smaller after one night of SD. These data suggested that SD did not affect the neuronal activation, but impaired the vasodilatory responses induced by either neuronal activation or hypercapnia.

To the best of our knowledge, this is the first human study to show that the blunted FV response under neuronal activation after SD was not due to impaired neuronal activity, and 24-h of SD reduced not only the neuronal activation-induced flow response, but also the hypercapnia-induced cerebral vasomotor reactivity. These data shed light on cerebral vascular changes caused by SD.

### Baseline flow velocity data before and after sleep deprivation

Baseline FV parameters were measured in the MCA before the breath-holding test and in the PCA at the end of the resting phase, before the beginning of the stimulation phase (Fig. 1). Although the baseline data before and after SD were not significant, there was a tendency in both middle and posterior cerebral arteries for higher resting flow velocities and lower PI after 24-h SD. The reduced PI after SD, which has never been reported before, reflects decreased vascular resistance (i.e., more dilated microvessels), leading to higher baseline flow velocities in the supplying arteries after SD compared with the control period. These data, indicating a tendency for vasodilation and higher cerebral blood flow after sustained brain activity caused by SD, are in line with human PET, MRI, and experimental optical data^10,15,30^. Braun et al. reported higher global CBF measured by PET in the awake period prior to sleep onset compared with the awake period after recovery sleep^30^. In a more recent study, Elvsåshagen et al.^15^ detected higher CBF in subjects after one night of SD than without SD using arterial spin labeling perfusion MRI. In addition to human studies, higher CBF was also reported in animal experiments^10^, showing increased oxygenated hemoglobin concentration during a 10-h SD in rats.

Although the neural mechanisms underlying sleep–wake cycle-dependent rCBF changes are not clear, and there are regional differences in CBF, a growing body of evidence supports that due to vessel expansion rCBF increases from morning to evening, but decreases after a night of sleep^9,10,15,30,31^. During sleep, brain metabolism decreases and provides an opportunity to restore the metabolism and to normalize the vessel tone to the baseline level^9,10^. Both human and animal studies showed that SD prevents the restorative processes, and sustained tissue activity is associated with further expansion of blood vessels and an increase in cerebral blood flow^9,10,15^.

### Blunted visual stimulation-evoked hemodynamic response after sleep deprivation

A tendency for higher absolute baseline FV values and lower absolute baseline PI in the PCA after SD suggested vasodilation of resistance vessels at the resting period in the sleep deprived participants. However, further decrease in absolute PI during visual stimulation, i.e. further vasodilation of resistance vessels, was smaller after SD than before SD (Table 2). These absolute PI data suggested that cerebral resistance vessels, which were more dilated in sleep deprived subjects in the resting phase, showed a smaller vasodilatory response during visual stimulation compared to the pre-SD, control period. However, changes in absolute FV values (either PSV or TAMV) in the PCA induced by visual stimulation were not significantly different before and after SD. As absolute FV values ​​show large interindividual variabilities and do not correlate with regional CBF, absolute FV parameters measured during the visual stimulation were normalized to baseline and expressed in relative values as a percentage of baseline. Using repeated measures ANOVA, analysis of the relative data showed that visual stimulation not only caused a smaller decrease in relative PI after SD (Fig. 3), but also that the increase in both relative TAMV and PSV was significantly smaller after SD than before SD (Fig. 2A, B). This observation indicates that neuronal activation induced a less pronounced vasodilation in the cerebral resistance vessels and less pronounced relative flow and FV increase in the PCA after 24 h of SD. This is well demonstrated in the figures (Figs. 2 and 3), and is obvious even if paired t-test revealed that only the maximum relative PSV and PI, but not the maximum relative TAMV changes were significantly different during visual stimulation before and after SD (Table 3.).

The blunted neuronal activity-evoked flow response observed in the present study is in line with other observations. Although Csipo et al. could not demonstrate a negative effect of SD on the vascular responses to cognitive stimulation or flicker light stimulation with TCD or retinal dynamic vascular analysis, respectively, they found a reduced hemodynamic response to finger tapping in the prefrontal and somatosensory cortices after SD when used fNIRS^16^. Similarly to our results, Schei and Rector^9^ and Phillips et al.^10^ observed cerebral vessel expansion, i.e., vasodilation of the resistance vessels in the resting period, and blunted auditory evoked hemodynamic response in rats during SD compared to the control condition with no SD. They concluded that SD with sustained brain activity requires higher CBF, which is provided by vessel expansion, leading to a decreased compliance of the vessels. It has been suggested that if vessels expand to the point where CBF can no longer increase, then there is a physical limit to nutrient delivery and waste removal^10^. Chronic exposure to sleep restriction in such a case can prevent the restorative function of sleep, leading to neural trauma. Our data also suggested that sustaind brain activity caused by one night of SD resulted in vasodilation of resistance vessels, which may decrease the vascular compliance and attenuate the further vasodilation under neuronal activation. In addition to this possible explanation for the reduced neuronal activation-induced flow response, other mechanisms of CBF dysregulation, which will be discussed in a later section, cannot be ruled out.

### Results of VEP and BHI

Latency and amplitude of VEP and breath-holding index were determined to separately assess neuronal activity and cerebral vasomotor reactivity. The amplitude of the visually evoked potential before and during SD was similar, indicating no major effect of SD on the occipital cortex activation. However, the latency of the P100 wave was larger during SD than before SD in our study, which was most probably due to fatigue. Similar to our findings, Hord and Tracy^32^ and Janocha et al.^33^ also described prolonged latency of evoked potentials in subjects with fatigue caused by 48-h of sleep loss or 2–3 days sleep insufficiency, respectively. However, others found that 24-h of SD had no effect on the latency of the P100 wave when using checkerboard stimulation, suggesting that SD did not affect the early visual processing^34,35^. As one of our new results, we showed reduced PCA flow velocity response caused by visual stimulation and no change in VEP amplitude after 24 h of SD suggesting that the impairment of the neurovascular coupling after SD was not due to impaired neuronal activity.

Measurement of the breath-holding index in the MCA showed that the cerebral vasomotor reactivity in response to hypercapnia was smaller during SD than before SD, suggesting that SD impairs the hypercapnia-induced vasodilation. It should be noted that there was a trend towards lower baseline PI and higher baseline TAMV not only in the PCA, but also in the MCA after SD, indicating vasodilation and reduced tone of the resistance vessels in the resting phase also in the MCA territory after SD. As the more dilated vessels of sleep deprived individuals have limited vascular compliance, they react less to a further vasodilatory stimulus, in this case, to hypercapnia. Another novel finding of the present study is that SD attenuates not only the neuronal activation-evoked flow response in the PCA, but also the cerebral vasomotor reactivity induced by hypercapnia in the MCA, which is independent of neuronal activation.

### Possible causes of decreased visual stimulation-evoked vascular response and reduced cerebral vasoreactivity during SD

Our results showed that SD impairs both neurovascular coupling in the PCA and cerebral vasomotor reactivity in the MCA. In line with our results, earlier studies suggested that sustained brain activity during SD requires higher cerebral blood flow that is provided by vasodilation of the cerebral resistance vessels^9,10^. The more dilated resistance vessels caused by SD may explain the reduced FV response under both neuronal activation (neurovascular coupling) and hypercapnia (cerebral vasoreactivity), because dilated resistance vessels have decreased compliance and react less to further vasodilatory stimuli. However, after SD, other mechanisms may also be considered as potential causes of the reduced vascular response to neuronal activation and hypercapnia.

Although vasodilation of cerebral resistance vessels is the final and essential step in both neurovascular coupling and cerebral vasomotor reactivity, they do not necessarily operate through the same regulatory mechanisms. However, earlier human studies have reported a reduced functional stimulation-evoked flow response under hypercapnia compared to normocapnia^36–38^, suggesting a partial overlap in the regulatory mechanisms responsible for neurovascular coupling and cerebral vasoreactivity. The flow response induced by neuronal activation is regulated by nitric-oxide, arachidonic acid metabolites, adenosine, metabolic products (CO_2_, lactate), potassium ion, and neurotransmitters including glutamate^21^, while hypercapnia affects CBF through pH, nitric-oxide and itself the pCO_2_^20^. Since both neuronal activation- and hypercapnia-induced vasodilations are at least partly mediated by the nitric oxide signaling pathway^39–44^, its impairment may interfere with the vasodilatory response required for both neurovascular coupling and cerebral vasoreactivity. Endothelial dysfunction^45–48^ and damage to the nitric-oxide signaling pathway^47–49^ was indeed observed during SD^45,46,49^ or insufficient sleep^47,48^, leading to a decreased vasodilatory response. Based on these results, in addition to resting vasodilation of the cerebral resistance vessels, CBF dysregulation due to altered nitric-oxide signaling pathway can also be assumed as a potential cause of impaired neurovascular coupling and cerebral vasoreactivity.

In the present study, no significant correlation was found between the visual stimulation-induced flow response in the PCA and the cerebral vasomotor reactivity in the MCA either before or after SD. This is not surprising, because there are not only common, but also different mechanisms in the regulation of the two processes. Moreover, the neuronal activation-induced flow response and the hypercapnia-induced cerebral vasoreactivity were evaluated in different arteries (PCA and MCA, respectively) in the present study, and different hemodynamic responses induced by hypercapnia were reported in different brain regions^50^. Nevertheless, our findings on reduced cerebral vasoreactivity may contribute to a better understanding of the pathomechanism of sleep deprivation-induced vascular changes, as it indicates that SD also impairs vasodilatory processes independent of neuronal activation.

### Potential risk of SD in terms of decreased vascular compliance

Phillips et al. suggested that if the vasodilation reaches its physical limit during chronic exposure to SD, metabolite delivery to the tissue and waste and heat removal might be impaired, leading to neuronal damage. During sleep, microvessels may regain their tone, and vascular compliance may recover.

In addition to the decreased vascular response to visual stimulation, the hypercapnia-induced vasodilation was also reduced after a 24-h SD in the current study. Although reduced, the BHI was within or close to the normal range in all subjects^51,52^, proving that after 24-h of SD, significant vasodilation could still be induced in the MCA territory by hypercapnia. This result indicated that this period of 24-h period of SD did not exhaust the cerebrovascular reserve capacity. However, since the regulation of CBF differs at least partly between the neurovascular coupling and the hypercapnia-induced flow response^47,53,54^, the significant reserve capacity observed under hypercapnia does not necessarily mean similar flow reserve during neuronal activation. Furthermore, it should be noted that our data are based on the study of young and healthy subjects after a relatively short period of SD, which cannot be applied to an older patient population with comorbidities and chronic sleep deficiency.

## Limitations

The present study has several limitations. First, we used TCD to assess cerebral hemodynamics, which has a poor spatial resolution and cannot detect regional differences. Moreover, absolute flow velocities show a huge interindividual variability and are not proportional to CBF in different individuals. Therefore, in the analysis, we also used relative flow velocity changes induced by a vasoactive stimulus, which correlate significantly with relative changes of CBF, supposing that the diameter of the insonated artery is not changed by the stimulus. Second, TCD examinations were performed twice: in the morning after a normal sleep and 24 h later, after a night of sleep deprivation. After the first examination, the TCD probes were removed, and mounted again 24 h later, which could result in altered angle of insonation. In order to avoid this methodological error, the site of the insonation was marked at the first examination, the same depth was used to measure the flow parameters, and always the strongest signal was searched and accepted. Moreover, relative flow velocities were also calculated, which are not sensitive to different insonation angles. Third, we measured the visual stimulation-induced flow velocity response in the PCA, and the hypercapnia-induced hemodynamic response in the MCA. Evaluation of vasomotor reactivity in the MCA was chosen, becuase normal BHI values are available for this artery. Since there are regional differences in the vasoreactivity^50^, direct comparison of the hypercapnia-induced flow velocity changes measured in the anterior circulation to the visual stimulation-evoked flow response assessed in the posterior circulation is not possible. Fourth, BHI was used to assess the cerebral vasoreactivity, which method has some pitfalls. To avoid an increase in intrathoracic pressure during breath holding, the volunteers were taught to perform the test without using the Valsalva manouvre. To eliminate variability in breath hold duration, each individual was asked to hold their breath for 30 s. Fifth, pCO_2_ or end-tidal CO_2_ was not measured before and after SD, and during BHI. It should be noted, however, that according to the literature data, spirometric and blood gas values did not differ before and after SD^55–57^. Finally, it should be noted that our volunteers were young and healthy subjects with no known comorbid conditions. Older patients with cerebrovascular risk factors may have a worse vascular status, so reduced vascular compliance caused by SD may cause more severe impairment in neurovascular coupling and cerebrovascular reactivity.

## Conclusion

Our data suggested that the impaired visually evoked FV response in the PCA (neurovascular coupling) after SD was not due to a decreased neuronal activation, but was most probably caused by a reduced vascular response to the occipital cortex activation. The decrease in cerebral vasomotor reactivity after SD showed that SD also has a negative effect on hemodynamic responses induced by other vasodilatory stimulus, independent of neuronal activation. The impaired vasodilatory response under neuronal activation and hypercapnia after SD could be due to vessel expansion caused by sustained brain activity, which may limit further dilation of the cerebral blood vessels, but other mechanisms of CBF dysregulation cannot be excluded either.

## Data availability statement

All data generated and analysed during this study are included in Supplementary Information files.

## Supplementary Information

Supplementary Information.
